# Supplementary material for: High Rate of Non-Human Feeding by Aedes aegypti Reduces Zika Virus Transmission in South Texas
Source: Viruses. 2020 Apr 17;12(4):453. doi: 10.3390/v12040453 (PMC7232486; doi:10.3390/v12040453)
Supplement: Supplementary file 1 [file viruses-12-00453-s001.pdf]

Supplemental Material

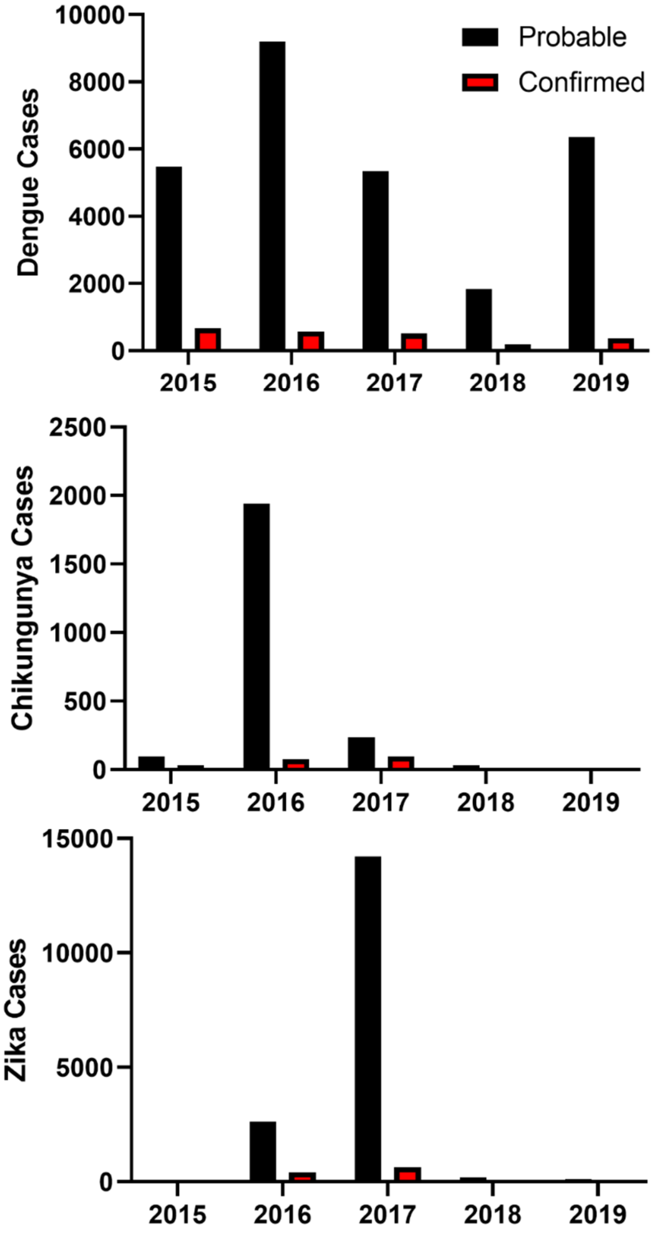

Figure S1: Probable and confirmed human cases of DENV, CHIKV and ZIKV from 2015 to 2019 in Tamaulipas, México. Probable cases also include those that were ultimately confirmed by PCR.

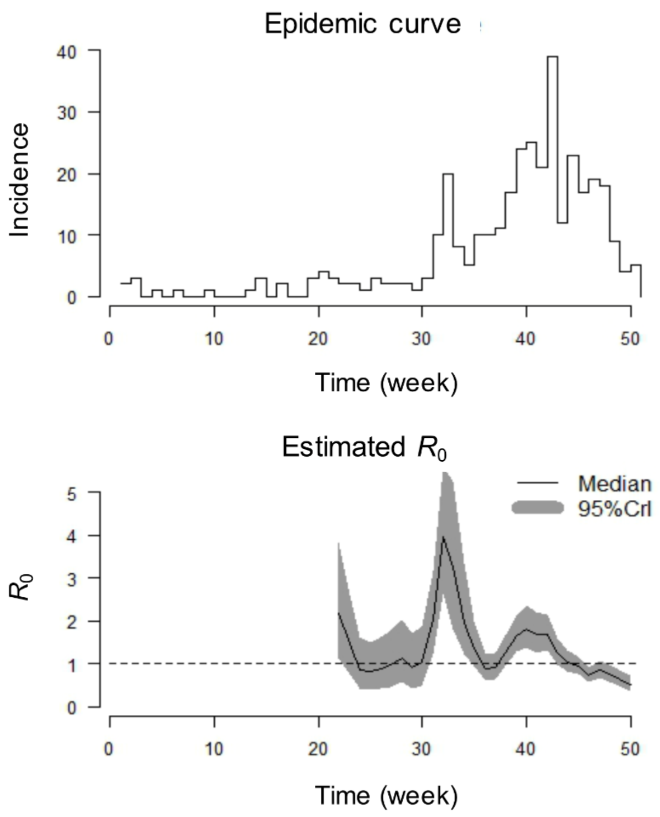

Figure S2: Weekly human Zika cases in Reynosa in 2017, and estimated effective reproductive number.

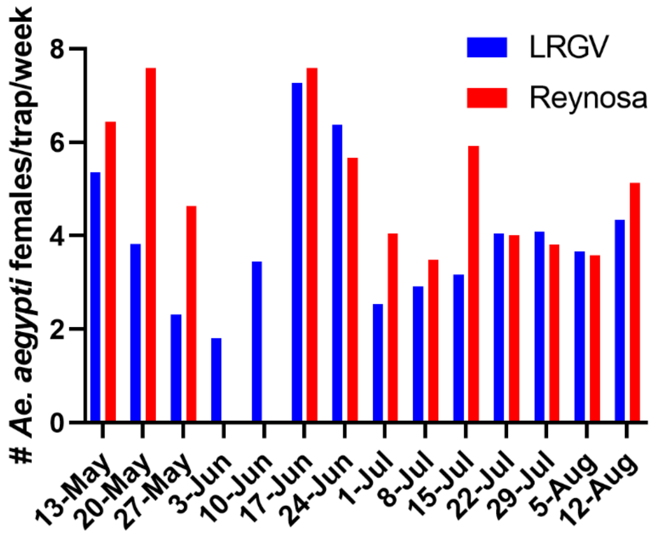

Figure S3: Weekly Autocidal Gravid Ovitrap (AGO) counts for *Ae. aegypti* in the Lower Rio Grande Valley (LRGV) and Reynosa between 13 May and 12 August, 2017.

Table S1. Vertebrate-specific primers used in this study.

Primer Name	Ratio	Sequence (5′–3′)	Direction	Ref.
VF1_t1	1	TGTAAAACGACGGCCAGTTCTCAACCAACCACAAAGACATTGG	Forward	[1,2]
VF1D_t1	1	TGTAAAACGACGGCCAGTTCTCAACCAACCACAARGAYATYGG	Forward	[1,2]
VF1i_t1	2	TGTAAAACGACGGCCAGTTCTCAACCAACCAIAAIGAIATIGG	Forward	[1,2]
VR1D_t1	1	CAGGAAACAGCTATGACTAGACTTCTGGGTGGCCRAARAAYCA	Reverse	[1,2]
VR1_t1	1	CAGGAAACAGCTATGACTAGACTTCTGGGTGGCCAAAGAATCA	Reverse	[1,2]
VR1i_t1	2	CAGGAAACAGCTATGACTAGACTTCTGGGTGICCIAAIAAICA	Reverse	[1,2]
BM1	1	CCCCTCAGAATGATATTTGTCCTCA	Forward	[3,4]
BM2	1	CCATCCAACATCTCAGCATGATGAAA	Reverse	[3,4]
Herp *	1	GCHGAYACHWVHHYHGCHTTYTCHTC	Reverse	[3,4]

* Use BM1 as forward primer.

Table S2. Universal invertebrate primers used in this study.

Primer Name	Ratio	Sequence (5′ -> 3′)	Direction	Reference
LCO 1490	1	GGTCAACAAATCATAAAGATATTGG	Forward	[5]
HCO 2198	1	TAAACTTCAGGGTGACCAAAAAATCA	Reverse	[5]

Table 3. Vertebrate densities resulting from community surveys.

	Community				Total	Proportion	95% CI	
	IHE	IHW	LP	TB			Lower	Upper
# Homes Surveyed	14	10	13	7	44			
Human	78	45	59	23	205	.382	0.342	0.424
Dog	31	49	66	22	168	.313	0.275	0.353
Cat	14	9	30	18	71	.132	0.106	0.164
Chicken	27	19	19	25	90	.168	0.138	0.202
Pig	0	3	0	0	3	.006	0.002	0.016

Table S4. Estimated vertebrate population densities based upon community surveys.

Community	Area (km^2)	Human	Dog	Cat	Chicken	Pig
				per km^2		
IHE	.33	5,209	2,046	930	1,767	0
IHW	.079	5,468	5,954	1,094	2,309	367
LP	.073	9,863	11,178	5,041	3,288	0
TB	.077	2,104	1,974	1,649	2,286	0
Total	.559	5,146	4,161	1,751	2,299	75
% of total vert. pop		38%	31%	13%	17%	0.6%

Table S5. Estimated number of homes, population sizes and area in the regions of the LRGV receiving mosquito sampling in the current study and Martin et al. 2019. We also present the number of bloodfed mosquitoes with host identification results from each community, how many unique homes had at least one specimen, and what proportion of blood meal results were human.

				Aedes aegypti			*Culex quinquefasciatus*		
Community	#Houses	GIS Pop (2010)	Area (km^2)	# of results	# of unique homes	Proportion human (n)	# of results	# of unique homes	Proportion human (n)
La Piñata	132	572	0.146	56	19	0.304 (17)	27	12	.037 (1)
Tierra Bella	47	191	0.074	22	10	0.318 (7)	6	5	0
Donna	122	510	0.115	8	3	0.250 (2)	-	-	-
Indian Hills W	124	337	0.076	14	9	0 (0)	-	-	-
Indian Hills E	311	1467	0.36	64	34	0.375 (24)	79	23	0
McAllen	67	227	0.116	3	2	0.333 (1)	1	1	0
Mesquite	39	162	0.039	2	2	0 (0)	2	1	0
Rio Rico	20	55	0.041	-	-	-	-	-	-
Donna Fig	49	154	0.042	2	2	0 (0)	1	1	0
Progresso	73	314	0.081	7	4	0.571 (4)	2	2	0
Christian Ct.	34	129	0.059	5	2	0.200 (1)	5	3	0
MCH Chapa	30	127	0.037	2	1	0.500 (1)	-	-	-
La Feria	70	222	0.056	1	1	1 (1)	-	-	-
Total	1118	4467	1.242	186	89		123	48	

References

1. Ivanova, N.V.; Zemlak, T.S.; Hanner, R.H.; Hebert, P.D. Universal primer cocktails for fish DNA barcoding. *Mol. Ecol. Notes* **2007**, *7*, 544–548.
2. Hernández-Triana, L.M.; Brugman, V.A.; Prosser, S.W.J.; Weland, C.; Nikolova, N.; Thorne, L.; De Marco, M.F.; Fooks, A.R.; Johnson, N. Molecular approaches for blood meal analysis and species identification of mosquitoes (Insecta: Diptera: Culicidae) in rural locations in southern England, United Kingdom. *Zootaxa* **2017**, *4250*, 67–76.
3. Hamer, G.L.; Kitron, U.D.; Goldberg, T.L.; Brawn, J.D.; Loss, S.R.; Ruiz, M.O.; Hayes, D.B.; Walker, E.D. Host selection by *Culex pipiens* mosquitoes and West Nile virus amplification. *Am. J. Trop. Med. Hyg.* **2009**, *80*, 268–278.
4. Medeiros, M.C.; Ricklefs, R.E.; Brawn, J.D.; Hamer, G.L. *Plasmodium* prevalence across avian host species is positively associated with exposure to mosquito vectors. *Parasitology* **2015**, *142*, 1612–1620, doi:10.1017/S0031182015001183.
5. Folmer, O., Black, M. , Hoeh, W., Lutz R., and Vrijenhoek, R. DNA primers for amplification of mitochondrial cytochrome c oxidase subunit I from diverse metazoan invertebrates. *Mol. Mar. Biol. Biotechnol.* **1994**, *3*, 294–299.
